# Cervical cancer prevention program in Mexico disrupted due to COVID-19 pandemic: Challenges and opportunities

**DOI:** 10.3389/fonc.2023.1008560

**Published:** 2023-03-09

**Authors:** Aurelio Cruz-Valdez, Lina Sofia Palacio-Mejía, Amado D. Quezada-Sánchez, Juan Eugenio Hernández-Ávila, Tatiana Galicia-Carmona, Lucely del Carmen Cetina-Pérez, Eder A. Arango-Bravo, David Isla-Ortiz, Carlos E. Aranda-Flores, Santos-Regino Uscanga-Sánchez, Vicente Madrid-Marina, Kirvis Torres-Poveda

**Affiliations:** ^1^Center for Population Health Research, Instituto Nacional de Salud Pública (INSP), Cuernavaca, Mexico; ^2^Consejo Nacional de Ciencia y Tecnología (CONACYT)—Instituto Nacional de Salud Pública (INSP), Cuernavaca, Mexico; ^3^Center for Evaluation and Surveys Research, Instituto Nacional de Salud Pública (INSP), Cuernavaca, Mexico; ^4^Department of Clinical Research and Medical Oncology, Instituto Nacional de Cancerología (INCAN), Mexico City, Mexico; ^5^Department of Oncology Gynecology, Instituto Nacional de Cancerología (INCAN), Mexico City, Mexico; ^6^Oncology Department, Hospital General de México “Eduardo Liceaga”, Mexico City, Mexico; ^7^Gynecologist Oncologist of MAGNI Gineco-Obstetras S.C, Mexico City, Mexico; ^8^Chronic Infections and Cancer Division, Center for Research on Infectious Diseases, Instituto Nacional de Salud Pública (INSP), Cuernavaca, Mexico

**Keywords:** COVID-19, uterine cervical neoplasm, prevention and control, health impact assessment, time series analysis, Mexico

## Abstract

**Introduction:**

The COVID-19 pandemic disrupted the preventive services for cervical cancer (CC) control programs in Mexico, which will result in increased mortality. This study aims to assess the impact of the pandemic on the interruption of three preventive actions in the CC prevention program in Mexico.

**Methods:**

This study is a retrospective time series analysis based on administrative records for the uninsured population served by the Mexican Ministry of Health. Patient data were retrieved from the outpatient service information system and the hospital discharge database for the period 2017–2021. Data were aggregated by month, distinguishing a pre-pandemic and a pandemic period, considering April 2020 as the start date of the pandemic. A Poisson time series analysis was used to model seasonal and secular trends. Five process indicators were selected to assess the disruption of the CC program, these were analyzed as monthly data (N=39 pre-pandemic, N=21 during the pandemic). HPV vaccination indicators (number of doses and coverage) and diagnostic characteristics of CC cases were analyzed descriptively. The time elapsed between diagnosis and treatment initiation in CC cases was modeled using restricted cubic splines from robust regression.

**Results:**

Annual HPV vaccination coverage declined dramatically after 2019 and was almost null in 2021. The number of positive Papanicolaou smears decreased by 67.8% (90%CI: -72.3, -61.7) in April–December 2020, compared to their expected values without the pandemic. The immediate pandemic shock (April 2020) in the number of first-time and recurrent colposcopies was -80.5% (95%CI:−83.5, −77.0) and -77.9% (95%CI: −81.0, −74.4), respectively. An increasing trend was observed in the proportion of advanced stage and metastatic CC cases. The fraction of CC cases that did not receive medical treatment or surgery increased, as well as CC cases that received late treatment after diagnosis.

**Conclusions:**

Our analyses show significant impact of the COVID-19 pandemic with declines at all levels of CC prevention and increasing inequalities. The restarting of the preventive programs against CC in Mexico offers an opportunity to put in place actions to reduce the disparities in the burden of disease between socioeconomic levels.

## Introduction

Cervical cancer (CC) is caused majorly by a persistent infection of high-risk human papilloma virus (HR-HPV). This neoplasm is considered preventable by HPV vaccination, for those cases caused by the genotypes included in the available vaccines and by routine screening for precancerous lesions. However, CC remains a significant cause of cancer-related mortality and a major public health problem, particularly in low- and middle-income countries (LMICs) (1). In 2020, GLOBOCAN estimated the occurrence of 604,127 new cases (particularly in middle-aged women) and 341,831 deaths from CC worldwide, with 80% occurring in LMICs (2). In 2020 9,439 new cases (4.8%) and 4335 deaths were estimated in Mexico, with a 5-year prevalence of 38/100,000, which is equivalent to 25026 prevalent cases (3).

In 2020, the World Health Assembly adopted a strategy for the elimination of CC, with the intention of achieving for all countries an incidence rate of less than 4 cases per 100,000 women by 2030 (4). For this, three strategies have been proposed: prevention (target of 90% of girls aged 15 years or younger fully vaccinated against HPV), early detection (target of 70% of women aged 35–45 years screened by molecular methods to detect HR-HPV DNA), and guaranteed treatment (target of 90% of women) diagnosed with CC.

According to the Mexican standard NOM 014-SSA2-1994 for the prevention, detection, diagnosis, treatment, control, and epidemiological surveillance of CC, a comprehensive control program for CC in Mexico must include all three types of prevention (5). Primary prevention includes specific protective actions through the free delivery of the anti-HPV prophylactic vaccine, in a two-doses vaccination schedule since 2016, as part of a universal program targeting girls in fifth grade of primary school, aged 9-11 years.

Secondary prevention includes early detection of premalignant cervical lesions in women aged 25–34 years either by a Papanicolaou (Pap) smear test or direct visualization with acetic acid when a Pap smear is not available, and biomolecular testing for HPV detection in women aged 35–64 years. These tests should be performed free of charge to all applicant women in public sector health facilities (5).Tertiary prevention includes the follow-up of women with premalignant lesions and CC, and timely treatment (6).

Latin America and the Caribbean (LAC) has been one of the areas most affected by the ongoing COVID-19 pandemic, with Mexico as an epicenter. Mexico has the fifth highest number of COVID-19 deaths in the world, after the United States, Brazil, India, and Russia (7). COVID-19 has affected, either directly or indirectly, all health systems in the world (8–10). Maternal and child health services, among other health services and programs, have reported poorer results and disruptions because of the pandemic (11, 12); sexual and reproductive health services (13, 14) and cancer screening programs have also been impacted (15).

Cancer screening programs worldwide, including CC preventive programs, have been particularly affected by the COVID-19 pandemic, as evidenced by lower HPV vaccination coverage rates (16, 17), fewer histological and cytological samples taken, fewer immunohistochemistry and molecular tests performed (18), fewer supplies available to perform molecular HPV laboratory services (19), a lower proportion of women screened for CC before and during the COVID-19 pandemic (20), excess CC diagnosis (21), and longer delays in treating cancer patients (22–24).

During the pandemic phase, Mexican national health authorities implemented hospital conversion strategies, prioritizing the allocation of human and biomedical resources for COVID-19 patients requiring hospitalization (25). Hospital conversion affected all “nonessential” health services, including public cancer screening programs. Further disruptions to these services came from reduced availability of public transportation, patient fear of going to hospitals, and staffing shortages, as some health workers were reassigned to support COVID-19 response services (26).

Since estimating the effect of COVID-19 on the resilience of health services depends on several assumptions, it is necessary to evaluate the effect of COVID-19 on primary, secondary and tertiary prevention strategies to compensate for opportunities lost due to the pandemic. Therefore, this study aims to assess the impact of the COVID-19 pandemic on the disruption of activities at all three levels of the CC prevention program in Mexico.

## Materials and methods

### Study design

This is a retrospective time series analysis based on administrative records to evaluate the disruption caused by the COVID-19 pandemic in the CC care program for uninsured population, served by the Mexican Ministry of Health (MoH). Administrative records of health services provided in outpatient and inpatient care in facilities managed by the MoH and clinical records of the National Cancer Institute (INCAN) were analyzed.

### Data source

Data were retrieved from the outpatient services information system (SIS) (27) and the hospital discharges database (SAEH) for the period 2017-2021. Both systems collect information on the part of the population lacking social security (approximately 50% of Mexico’s total population) that received medical care in health facilities administered by the MoH. Anonymized data on patients who attended INCAN in the period 2017–2021 were also included. Approval was obtained for access to clinical record information (INCAN/CI/O411/O411/2022/082). INCAN is one of the main oncology referral centers in Mexico, managed by the MoH and serving the uninsured population. Data were aggregated by month; pre-pandemic and pandemic periods were identified, with April 2020 as the start date of the pandemic. Thus, the pre-pandemic period was defined as January 2017 to March 2020 (39 months), and the pandemic period as April 2020 to December 2021 (21 months).

### Measured indicators

Data retrieved from the SIS included HPV vaccination data (primary prevention): vaccination, coverage, first and second doses of HPV vaccine in female students in grade 5 and/or 11 years of schooling, and third dose of HPV vaccine in females aged 14 years and older; data on screening and treatment of precancerous lesions (secondary prevention): total cytology read, positive cytology, first-time colposcopy, and recurrent colposcopy; diagnosis and treatment of invasive cancer (tertiary prevention): CC-related hospital discharges according to ICD-10 leading cause classification. Indicators of the level of tertiary prevention retrieved from the INCAN database included: histopathological diagnosis, clinical stage, type of treatment and time from diagnosis to initiation of treatment.

Five process indicators were selected to evaluate the disruption on the CC care program: total Pap smears read, positive Pap smears, first-time colposcopy, and subsequent colposcopy as indicators of the secondary prevention level, and CC-related hospital discharges according to ICD-10 classification of main cause as indicators of the tertiary prevention level. In Mexico, HPV vaccination is massively administered during the National Health Weeks; thus, these indicators of vaccine administration could not be included in our statistical models; instead, the results of a descriptive analysis are given in the corresponding section. Data on tertiary prevention retrieved from the INCAN database were aggregated by pre-pandemic and pandemic periods and by month to calculate average time between diagnosis and treatment initiation.

### Statistical analysis

A descriptive analysis of all variables was performed; frequencies, percentages, and central tendency and dispersion statistics were calculated, and data were plotted to capture trends. We fitted Poisson regression models using each of the five process indicators as dependent variable. These models were fitted with Newey-West standard errors, which are consistent in terms of heteroscedasticity and autocorrelation (28). Our linear predictor, the logarithm of the mean in a Poisson regression model, included a linear time term and indicator variables for each month of the year (one excluded as a reference category) to model seasonal fluctuations around the trend. The trend model coefficient was expressed as the mean ratio between consecutive months after exponentiation, including a 95% confidence interval. Our models were trained from January 2017 through March 2020, extending the trend to include a seasonality component from April 2020 through December 2021, to estimate expected monthly values for each process indicator under the model and during the pandemic period; these were used to contrast observed values.

The sum of expected values for each of the five process indicators was calculated for four periods: April 2020, April-December 2020, January-December 2021, and April 2020 to December 2021. Standard errors for periods longer than a month were obtained through the delta method, to generate 95% confidence intervals (29). The differences between observed and expected values were then calculated, and confidence limits were mapped from those obtained for expected values to the differences. The differences were also calculated as percentages with respect to the expected values. In this framework, the analyzed process indicators in the pandemic period were considered as realized observations, rather than random variables; therefore, the inference problem focused on estimating baseline values that reproduced a pre-pandemic environment and approximated a counterfactual to the pandemic. A similar approach has been previously applied to estimate excess deaths from different causes in Mexico (30).

The time (number of days) elapsed from diagnosis to treatment initiation from INCAN patient records was modelled as a function of the calendar time (year and month) of patient records using robust regression. Predictors included monthly indicator variables (except for a reference category) to capture possible seasonal fluctuations, and a 4-knot restricted cubic splines function with respect to monthly time (31, 32).

## Results

### Impact on HPV vaccination (primary prevention)

**Figure 1:**
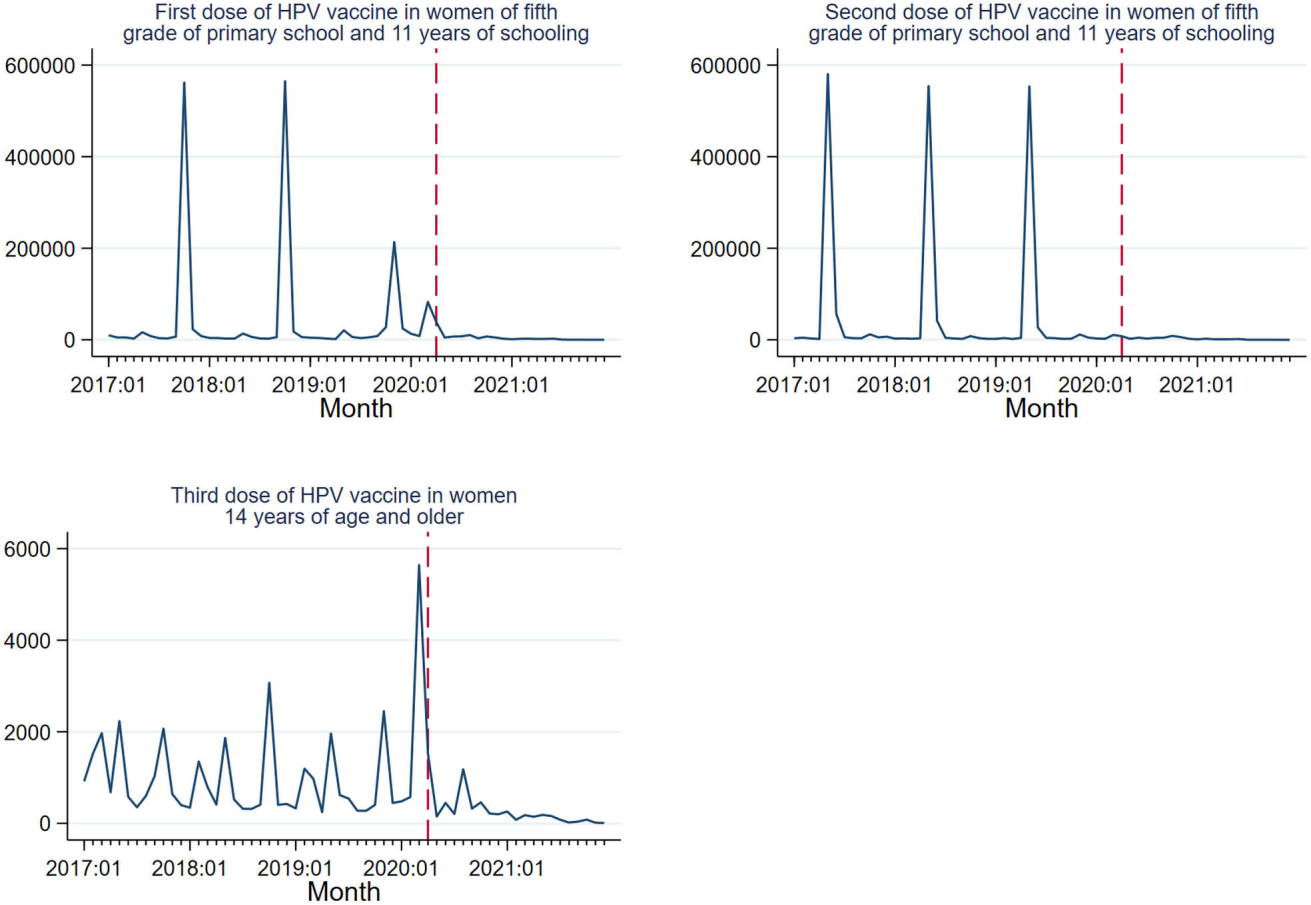
HPV vaccination trends 2017–2021. Mexico. Source: Outpatient provided services information system. 2017-2021. DGIS.

HPV vaccination is administered during National Health Weeks in October or November each year. The observed patterns from 2017 to 2021 are shown in **Figure 1**, with a significantly reduced vaccine delivery in 2019 compared to previous years. HPV vaccination dropped to near zero after April 2020. This pattern was also evident for the second dose, with no reduction in 2019. The third dose in risk population, in contrast, is delivered throughout the year (**Figure 1**), although significantly fewer vaccines were applied after April 2020. With respect to HPV vaccination coverage rates in the target population, showed a significant decline for the first dose of 59% in 2018 to 30.1% in 2019, 17.8% in 2020 and 1.2% in 2021.

### Impact on screening and treatment of precancerous lesions (secondary prevention)

Monthly descriptive statistics for selected program indicators are shown in **Table 1**. In general, the number and variability of services provided were lower during the pandemic than in the pre-pandemic months, except for CC hospital discharges, which showed similar descriptive statistics in both periods.

**Table 1:** Descriptive statistics of monthly data of selected indicators of the CC program.

Period	*Observed months (N)*	Monthly mean (SD)	P50 (P25, P75)	Min-Max
Pap smears				
Pre-pandemic	39	42901 (15215)	41867 (31252, 53720)	14934-75683
Pandemic	21	19779 (6477)	21180 (13633, 24662)	8413-31380
Positive pap smears				
Pre-pandemic	39	2836 (701)	2829 (2499, 3256)	1158-4386
Pandemic	21	1691 (728)	1909 (1070, 2070)	553-2988
First-time colposcopies				
Pre-pandemic	39	4279 (748)	4350 (3667, 4772)	2735-5622
Pandemic	21	1872 (879)	1956 (1116, 2736)	598-3385
Recurrent colposcopies				
Pre-pandemic	39	14130 (2250)	13655 (12675, 15991)	9881-19268
Pandemic	21	4942 (1706)	4920 (3718, 5804)	1973-7749
Cervical cancer hospital discharges				
Pre-pandemic	39	867 (145)	861 (770, 925)	562-1226
Pandemic	21	914 (129)	954 (829, 975)	670-1228

The pre-pandemic period is defined as January 2017–March 2020 (N=39 months); the pandemic period is defined as April 2020–December 2021 (N=21 months). Descriptive statistics of monthly counts are shown: mean, standard deviation (SD), median (P50), 25^th^ and 75^th^ percentiles (P25, P75), minimum (Min) and maximum (Max).

Source: Outpatient provided services information system and hospital discharges. 2017–2021. DGIS.

**Figure 2:**
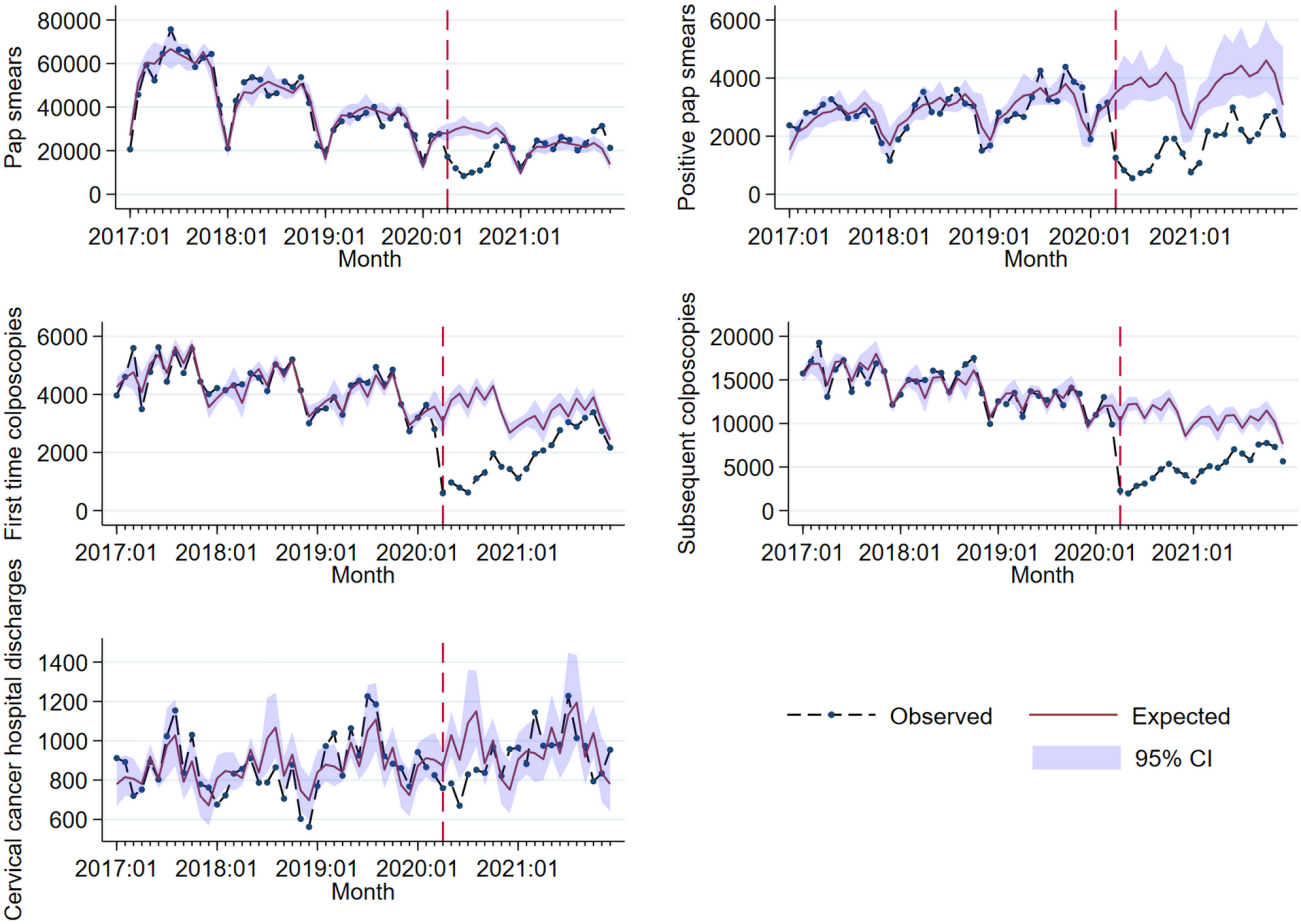
Observed and fitted trends for five process indicators to evaluate disruption of the CC program. Source: Outpatient provided services information system and Hospital discharges. 2017-2021. DGIS. The dashed line marks the start of the pandemic period (April 2020–December 2021). Expected trends were obtained from Poisson models trained during the pre-pandemic period (January 2017–March 2020). The linear predictor included a time linear trend and month indicator variables to model seasonality. Standard errors were heteroskedastic and autocorrelation consistent using the Newey-West methodology with two lags. CI, Confidence Interval.

Observed counts of process indicators related to CC program services along with their differences with respect to their expected values under a pre-pandemic trend are shown in **Table 2** for four pandemic periods: a) an immediate shock in April 2020, b) April to December 2020, c) January to December 2021, and d) April 2020 to December 2021. At the beginning of the pandemic period in Mexico (April 2020), the number of Pap smears showed an abrupt reduction of 38.0% (**Table 2**), followed by a slow upward trend (**Figure 2**). Results from the Poisson time-series model that adjusted for seasonal fluctuations showed a negative slope during the pre-pandemic period with a monthly reduction of 2.1% (mean ratio [MR] = 0.979, 95% CI: 0.976–0.982) in the number of Pap smears, this corresponds to an annual reduction of 22.5%. Overall, the number of Pap tests decreased by 44.1% in 2020 (April–December) with respect to the number expected as predicted by our model. The number of Pap smears recovered in 2021, exceeding by 12.6% our model predictions. In the period April 2020–December 2021, an overall reduction of 16.1% was observed (**Table 2**).

**Table 2:** Estimation of changes in secondary prevention services and CC hospital discharges in the pandemic period with respect to pre-pandemic projected trends.

Indicator	Period analyzed	Observed	Expected (95%CI)	Difference observed minus expected	
				As a count (95%CI)	As percentage (95% CI)
Pap smears	Apr. 2020	17279	27871 (23924, 32470)	−10592 (−15191, −6645)	−38.0 (−46.8, −27.8)
	Apr. to Dec. 2020	140178	250772 (229596, 271949)	−110594 (−131770, −89418)	−44.1 (−48.5, −38.9)
	Jan. to Dec. 2021	275182	244301 (216674, 271928)	30881 (3254, 58508)	12.6 (1.2, 27.0)
	Apr. 2020 to Dec. 2021	415360	495073 (446497, 543649)	−79713 (−128289, −31137)	−16.1 (−23.6, −7.0)
Positive pap smears	Apr. 2020	1258	3484 (2900, 4187)	−2226 (−2929, −1642)	−63.9 (−70.0, −56.6)
	Apr. to Dec. 2020	10713	33320 (27972, 38668)	−22607 (−27955, −17259)	−67.8 (−72.3, −61.7)
	Jan. to Dec. 2021	24795	45455 (36300, 54610)	−20660 (−29815, −11505)	−45.5 (−54.6, −31.7)
	Apr. 2020 to Dec. 2021	35508	78775 (64305, 93245)	−43267 (−57737, −28797)	−54.9 (−61.9, −44.8)
First-time colposcopies	Apr. 2020	598	3063 (2594, 3616)	−2465 (−3018, −1996)	−80.5 (−83.5, −77.0)
	Apr. to Dec. 2020	10301	32844 (30790, 34898)	−22543 (−24597, −20489)	−68.6 (−70.5, −66.5)
	Jan. to Dec. 2021	29020	39158 (35933, 42382)	−10138 (−13362, −6913)	−25.9 (−31.5, −19.2)
	Apr. 2020 to Dec. 2021	39321	72001 (66761, 77242)	−32680 (−37921, −27440)	−45.4 (−49.1, −41.1)
Recurrent colposcopies	Apr. 2020	2270	10293 (8881, 11930)	−8023 (−9660, −6611)	−77.9 (−81.0, −74.4)
	Apr. to Dec. 2020	32638	101766 (96147, 107385)	−69128 (−74747, −63509)	−67.9 (−69.6, −66.1)
	Jan. to Dec. 2021	71153	122323 (113649, 130997)	−51170 (−59844, −42496)	−41.8 (−45.7, −37.4)
	Apr. 2020 to Dec. 2021	103791	224089 (209989, 238189)	−120298 (−134398, −106198)	−53.7 (−56.4, −50.6)
Cervical cancer hospital discharges	Apr. 2020	759	873 (800, 953)	−114 (−194, −41)	−13.1 (−20.4, −5.1)
	Apr. to Dec. 2020	7476	8489 (7629, 9348)	−1013 (−1872, −153)	−11.9 (−20.0, −2.0)
	Jan. to Dec. 2021	11721	11600 (10165, 13035)	121 (−1314, 1556)	1.0 (−10.1, 15.3)
	Apr. 2020 to Dec. 2021	19197	20089 (17819, 22358)	−892 (−3161, 1378)	−4.4 (−14.1, 7.7)

Expected values were predicted from Poisson regression models trained during the pre-pandemic period, January 2017–March 2020. The linear predictor included a time linear trend and month indicator variables to model seasonality. Standard errors were heteroskedastic and autocorrelation consistent using the Newey-West methodology with two lags. Source: Outpatient provided services information system. 2017-2021. DGIS.

With respect to pre-pandemic data, positive results of Pap smears showed an upward trend, although a reduction of 63.9% was observed at the beginning of the pandemic (April 2020). A reduction of 67.8% was found in positive Pap smears in the period April–December 2020, compared with the value predicted by our model. The overall reduction in 2021 was 45.4%, and it was 54.9% in the period April 2020–December 2021 (**Table 2**).

The number of first-time and recurrent colposcopies showed a downward trend in the pre-pandemic period after adjusting for seasonality; on average, there was a monthly decrease of 0.7% (MR = 0.992, 95% CI: 0.990–0.994) in the number of first-time colposcopies, and a decrease of 0.9% (MR = 0.990, 95% CI: 0.989–0.993) in the number of recurrent colposcopies, corresponding to an average annual reduction of 9.1% and 10.6%, respectively. The number of first-time and recurrent colposcopies also showed a sudden reduction of 80.5% and 77.9%, respectively, in April 2020, followed by an upward trend (**Table 2**; **Figure 2**).

Despite this recovery trend, the number of first-time and recurrent colposcopies was below the expected values throughout the pandemic period, as shown in **Table 2**. A reduction of 68.6% was observed in the number of first-time colposcopies in 2020, and of 25.9% in 2021, while the number of recurrent colposcopies was lower than the expected value by 67.9% in 2020 and by 41.8% in 2021. During the entire pandemic period, the number of first-time and recurrent colposcopies was lower than expected according to our model by 45.4% and 53.7%, respectively (**Figure 2**).

### Impact on diagnosis and treatment of invasive cancer (tertiary prevention)

The number of CC-related hospital discharges also showed a reduction at the beginning of the pandemic period, albeit to a lesser extent (13.1%), followed by a recovery trend in 2020 and 2021 (**Table 1**; **Figure 2**). An overall reduction of 11.9% was observed in 2020 with respect to expected hospital discharges in our model. In 2021, no significant differences were observed in the number of hospital discharges with respect to our model in the absence of a pandemic shock.

The distribution of categories of tertiary prevention indicators from patient data of the INCAN database are described in **Table 3**. An analysis of the number of histopathological diagnoses during the pandemic showed a slight decrease in squamous (from 83.6% to 82.3%) and adenosquamous (from 2.2% to 1.7%) diagnoses compared to the pre-pandemic period, and a small increase in adenocarcinomas (from 10.7% to 11.4%) and neuroendocrine involvement (from 1.6% to 1.9%). While a higher proportion of women were diagnosed in an early stage (21.6%) pre-pandemic; this proportion dropped to 11.4% during the pandemic. A slight increase in the proportion of locally advanced stages was also observed, from 61.8% to 63.6%, while the fraction of metastatic patients increased from 16.6% to 24.5%. The occurrence of untreated cases increased by 3.2 percentage points in the pandemic compared to the pre-pandemic period, and the percentage of women who had access to surgical treatment decreased from 21.1% to 11.7%. With respect to the time elapsed from diagnosis to treatment initiation, the percentage of women who were cared for immediately after diagnosis increased in the pandemic from 5.2% (pre-pandemic) to 8.4%, as did those who were treated within 1-2 weeks. Overall, the fraction of women seen within 2 weeks increased from 6.6% to 9.9%; however, the proportion who received treatment after 8 weeks increased from 36.6% to 46.6% (**Table 3**).

**Table 3:** Indicators of the tertiary prevention level from INCAN database.

		Pre-pandemic *N* = 1125	During pandemic *N* = 464	Overall *N* = 1589
Histopathological diagnosis				
Squamous cell carcinoma		940 (83.6%)	382 (82.3%)	1322 (83.2%)
Adenocarcinoma		120 (10.7%)	53 (11.4%)	173 (10.9%)
Adenosquamous carcinoma		25 (2.2%)	8 (1.7%)	33 (2.1%)
Neuroendocrine carcinoma		18 (1.6%)	9 (1.9%)	27 (1.7%)
Other		22 (2.0%)	12 (2.6%)	34 (2.1%)
Clinical stage				
Early		243 (21.6%)	50 (11.4%)	294 (18.5%)
Locally advanced		695 (61.8%)	278 (63.6%)	992 (62.4%)
Metastatic		187 (16.6%)	107 (24.5%)	301 (18.9%)
Not specified		0 (0%)	2 (0.5%)	2 (0.1%)
Type of treatment				
None		58 (5.2%)	39 (8.4%)	97 (6.1%)
Surgical		237 (21.1%)	52 (11.2%)	289 (18.2%)
Concomitant radio-chemotherapy		689 (61.2%)	288 (62.1%)	977 (61.5%)
Chemotherapy		141 (12.5%)	85 (18.3%)	226 (14.2%)
Time from diagnosis to start of treatment				
Immediate		58 (5.2%)	39 (8.4%)	97 (6.1%)
1–2 wk		16 (1.4%)	7 (1.5%)	23 (1.4%)
3–4 wk		108 (9.6%)	32 (6.9%)	140 (8.8%)
5–6 wk		236 (21.0%)	70 (15.1%)	306 (19.3%)
7–8 wk		295 (26.2%)	100 (21.6%)	395 (24.9%)
> 8 wk		412 (36.6%)	216 (46.6%)	628 (39.5%)

Observations are patient data during the pre-pandemic (N=1125) and the pandemic (N=464) periods.

Source: INCAN database.

**Figure 3:**
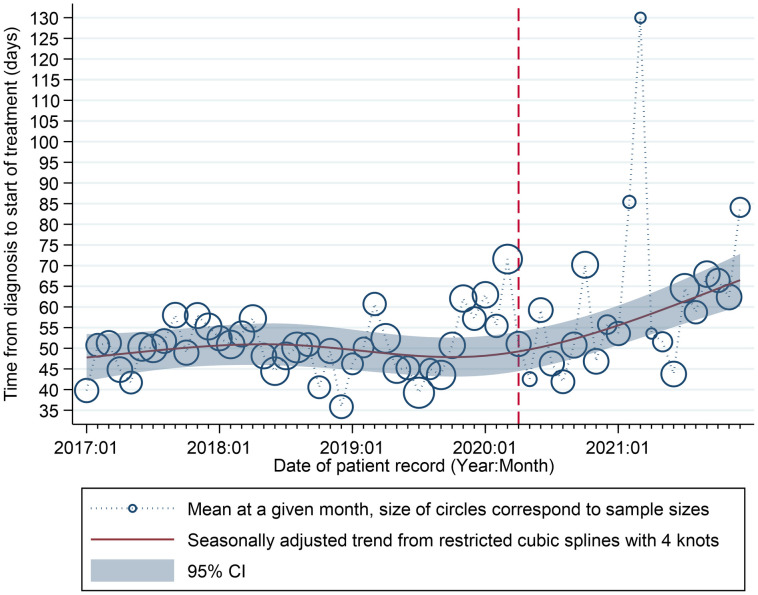
Time elapsed from diagnosis to treatment initiation (days) and date of patient records. Source: INCAN Database Relationship between time from diagnosis and start of treatment. The data points show the average time in days between diagnosis and start of treatment for each month in the INCAN database from January 2017 to December 2021 and the solid line are the fitted values from robust regression model. The dashed line marks the start of our defined pandemic period.

A robust regression analysis of the average time elapsed between diagnosis and treatment initiation (in days) as a function of the date of patient records is shown in **Figure 3**. The restricted cubic spline clearly shows an increasing trend in the time between diagnosis and initiation of treatment after April 2020, even though fewer patients were received. The point size of each data point is proportional to the number of patients.

## Discussion

The COVID-19 pandemic has disrupted CC screening programs around the world. This study assesses the impacts of the COVID-19 pandemic on CC prevention program in Mexico. It is important to highlight that given the limited availability of data sources and their quality, we organized effects into three levels considering the following indicators: first, the number of HPV vaccine doses and coverage in the target population (primary prevention); second, the number of positive Pap smears and, and the number of initial and subsequent colposcopy examinations (secondary prevention); third, the number of discharges associated with CC, histopathological diagnosis, clinical stage, type of treatment and time from diagnosis to initiation of treatment (tertiary prevention).

Overall, our analyses revealed significant declines in all levels of CC prevention. In primary prevention, the sharp decline in HPV vaccine doses delivered to the target populations between 2020 and 2021 coincides with the COVID-19 pandemic and the shortage of HPV vaccines on the global market due to an increased demand for such vaccines to achieve the elimination of CC as global target (33) In Mexico, this led to a temporary suspension of vaccination efforts and delayed interventions for the 2020, 2021, and 2022 target populations.

According to the WHO/UNICEF report, we found that vaccination was almost nil in 2021 (34). The COVID-19 pandemic has limited access to vaccines (not just against HPV) in many low- and middle-income countries (35). It is unclear whether the vaccine production capacity will meet global demand. Countries that have discontinued routine HPV vaccination can start plans for future catch-up campaigns for young people who missed HPV vaccination during the pandemic. However, the Strategic Advisory Group of Experts on Immunization (SAGE) Committee proposed strategies to address vaccine shortages during this period, including pausing vaccination of older women (> 15 years), establishing multi-age cohorts until sufficient supplies are available to meet global demand and protocols for delaying the second dose of vaccine by 2-3 years (33).

Two previous studies documented the impact of the COVID-19 pandemic on HPV vaccination services. Estimates of the impact of HPV vaccine coverage during the pandemic in the United States reported that coverage declined in March and April 2020, reaching a low of 23% in previous years (17). Another study reported a significant reduction in the average dose of HPV vaccine administered in Brazil from April to September 2019 (16). Many low- and middle-income countries had to delay of introduction of HPV vaccination (35).

In Mexico, as in other LAC countries, efforts to immunize target populations should be pursued in the months and years ahead in order to restore HPV vaccination rates and minimize medium-term consequences (36). In the long term, HPV vaccines accessibility will improve with the development of new, cheaper and faster-to- manufacture vaccines, which are expected to become available in the next few years (35). One government measure is to administrate only one dose of HPV vaccine. It is well known that one dose of HPV vaccine suffices to achieve good HPV serum antibody levels in girls under 12 years of age and is recommended (37). Therefore, this measure suffices to protect the target population of this group of HPV vaccines.

Regarding secondary prevention, the only indicators available for analysis were the number of analyzed/positive Pap smears and the number of initial and subsequent colposcopy examinations. It is important to note that although cervical screening in Mexico includes both cytology and molecular HPV testing, only information on cytology results is available.

Successful secondary prevention of CC is a multistep process that includes screening of the target population, triage of positive results, colposcopy-biopsy to confirm cervical precancer, and treatment of the precancer. Although limited, comparing the number of Pap smears and colposcopies performed during the pandemic with previous periods allows us to estimate the impact of disruptions of CC prevention program.

The number of positive Pap smears dropped at the start of the pandemic a tends to recover in 2021. This recovery coincides with the application of specific technical guidelines and protocols to reduce the risk of contracting COVID 19 issued by national health authorities (38). In Mexico, only one previous study has performed a similar analysis using data from various health services from the Mexican Institute Social Security (IMSS) Health Information System including the number of women screened for CC between January 2019 and December 2020, this indicator fell by 68% (39).

In an international context, the significant decline in cervical cancer screening rates was due to lockdowns and travel restrictions to contain the COVID-19 pandemic (20). In a study conducted in England, a 6.4% deficit was observed in the number of screening samples with respect to expected values before the pandemic (21).

The overall reduction in the number of Pap smear tests during the pandemic in 2020 with respect to historical data found herein (41.1%) is similar to that reported in Belgium (43.3%) (18) but lower than in Scotland (56%) (40), Italy (64.5%) (41), California (78%) (42), the United States (84%) (43), Canada (85%) (44), and Slovenia (92%) (45).

While our results show that the number of colposcopy procedures performed in Mexico was already declining in the pre-pandemic era, the number of first and recurrent colposcopy procedures decreased further by 45.4% and 53.7% in 2020–2021. A Canadian study reported an average monthly reduction of 39.7% in colposcopy volumes from March to August 2020 compared with the same period in 2019 and a 75.1% reduction at the onset of the pandemic (44). However, other studies that did not provide numerical data reported that colposcopy in women with minor or low-grade cytological abnormalities or persistent HPV infection was delayed worldwide because of the pandemic (40).

According to information from Mexico’s Unified Epidemiological Surveillance System, the impact of the COVID-19 pandemic on CC care programs was reflected in the lower number of reported cases of intraepithelial lesion in 2020 (46). We observed a slight recovery in 2021, but the number of cases was still below those reported in 2019. On the other hand, there are no official reports showing the impact of COVID-19 on indicators included in the regulatory documents related to screening coverage, diagnosis, evaluation and treatment of intraepithelial lesions, and efficiency in access to health services.

At the level of tertiary prevention, according to INCAN data, the most frequently diagnosed type of CC in Mexico before the pandemic was squamous cell carcinoma. Unfortunately, the pandemic there was an increase in the number of cases diagnosed at locally advanced and—even worse—metastatic stages. This result is consistent with a comparative analysis of three independent cervical models by the Cancer Intervention and Surveillance Network of the National Cancer Institute, which found that COVID-19-associated disease would cause a small net increase in the number of CC cases by 2027 (47).

Among the most relevant indicators for evaluating tertiary prevention efforts are the proportion of diagnosed CC cases that received treatment and the estimated time to initiate therapy (48). Our analysis shows that between 2020 and 2021, the proportion of untreated CC confirmed cases increased, while the proportion of early-stage CC cases treated with surgery decreased, becoming the scenario predicted by expert groups in hospitals with significant burden of COVID-19 cases (49).

The meantime between the diagnosis of CC and initiation of treatment has increased since the start of the pandemic. Most patients started treatment after eight weeks, and the relative numbers of this group have continued to increase during the pandemic. Evaluation of this indicator is important in Mexico, as the population most affected by COVID-19-related mortality lives in overpopulated and poor areas (50) and is also the most affected by CC (51).

This delay in treatment because of the pandemic has been badly documented, and few studies have described the impact of treatment delay on survival in patients with early-stage CC (22–24). A US study reported that the average wait time from CC diagnosis to hysterectomy was 4 weeks, and longer waiting times of 2 to 12 weeks were associated with an increased risk of all-cause mortality (23). Since the designated treatment procedures for radical hysterectomy in early-stage CC require hospitalization, these procedures will have to be postponed in areas with higher hospital demand (49).

In our study, it was not possible to analyze survival because the follow-up times of patients during the pandemic are still very short. According to a study on survival of delaying the initiation of concurrent chemoradiotherapy in women with locally advanced CC, in the absence of factors related to tumor aggressiveness, a short waiting time for treatment initiation (<10 weeks) may not be associated with an increased risk of mortality in women with this type of cancer (24). This study is relevant in the context of the current COVID-19 pandemic because fluctuating waves of infection force highly specialized hospitals to significantly reduce access to oncology care services, which would mean a delay in the treatment of these patients.

This study identified several areas for improvement at each sub-process or level of CC prevention program in Mexico, which have been affected by disruptions of preventive health and cancer care services because of the COVID-19 pandemic, in order to achieve the WHO goal of eliminating CC by 2030. Considering the cost of human life and suffering, implementing proper management of CC prevention programs should be a top priority for decision-makers and policy makers of CC prevention systems in all LAC countries to improve the performance indicators (52, 53), owing to the high prevalence of CC, the female cancer with the greatest preventive potential given its natural history.

The WHO global strategy to promote the elimination of CC as a public health issue proposes three coverage targets to be achieved (4): HPV immunization coverage in 15-year-old girls (70%); cervical screening coverage in women aged 35–45 years (70%), at least once in lifetime screening with valid evidence; and treatment coverage in women with precancerous lesions or CC (90%).

From 2017 to 2021, coverage of LAC complete HPV immunization schedule remains low (54). In the region, about 74% of women aged 30–49 years have been screened for cervical cancer at least once in their lifetime (55). The reported full coverage of the HPV vaccination program in Mexico was 97%, 96%, 95%, 5%, 5%, and 1% in 2017, 2018, 2019, 2020, and 2021, respectively (34). Screening coverage in 2019 exceeded 50% of the target population for that year. Although diagnostic screening coverage in colposcopy clinics exceeded 50% in 2019, there is no reliable information on the proportion of patients treated within 92 calendar days (56).

**Figure 4:**
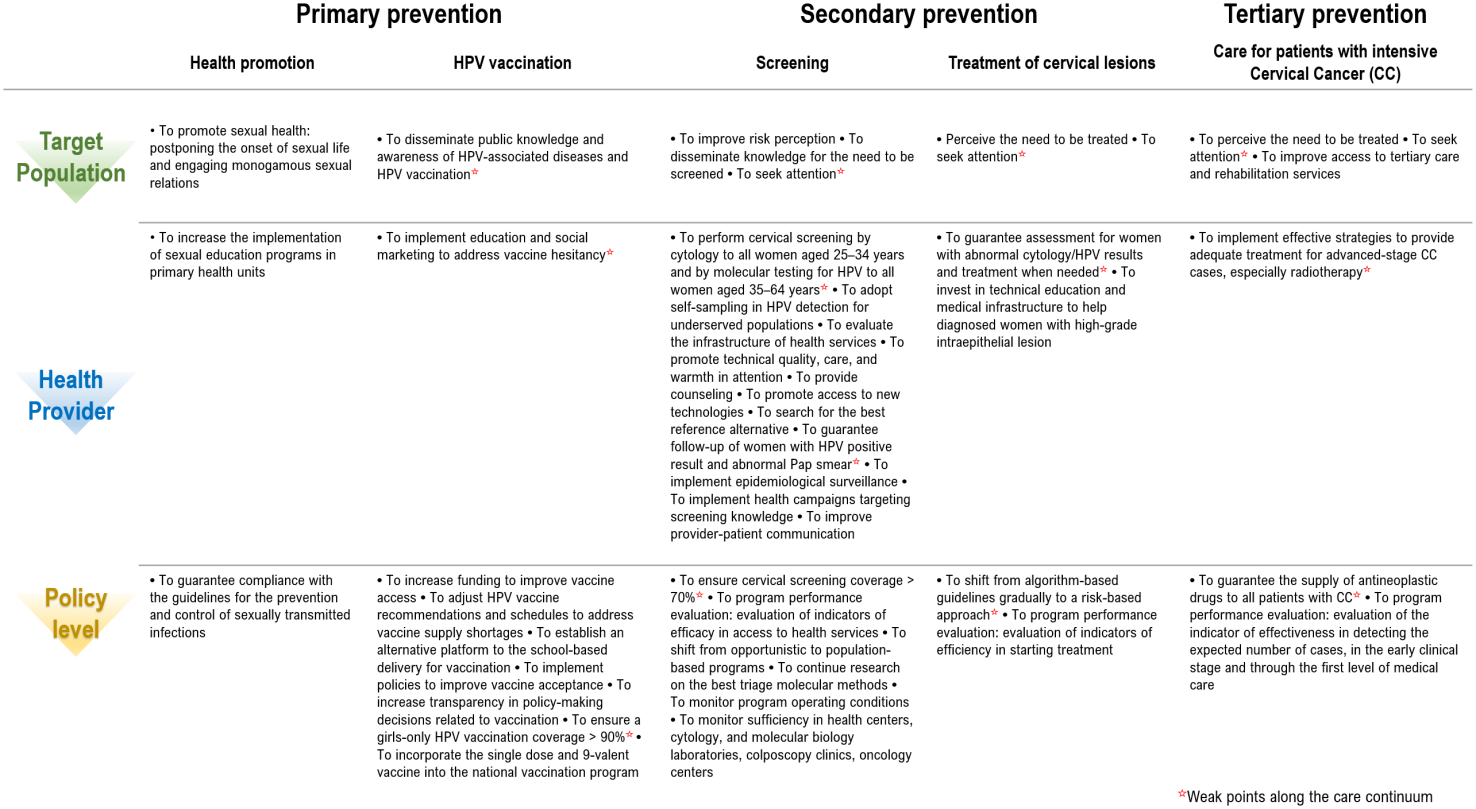
Process model of the cervical cancer prevention program in Mexico. The proposed strategies should be considered by health care providers and policymakers along the cascade of care to move toward the goal of eliminating cervical cancer in Mexico, as well as the critical control points in Mexico’s cervical cancer prevention program process model. ^*^Weaknesses along the continuum of care.

A disadvantage of multistep prevention programs is the need for multiple patient visits, including screening, colposcopy, treatment, and surveillance. At each step, there is a possibility that follow-up could be lost because of factors related to the patient, provider or health system, and an untreated precancerous lesion could progress to cancer (57). In this context, we suggest strategies to improve the prevention, diagnosis, and timely treatment program of CC in Mexico and to achieve the goal of CC elimination at the preventive level through their respective components, as well as three main actors for implementing the program, as shown in **Figure 4**.

Our study has strengths and limitations. The main strength and contribution of this work is that it allowed us to identify areas affected by the impact of the pandemic in order to improve CC prevention program and provide recommendations for policymakers involved in the management of this program in Mexico towards the goal of CC elimination.

The main limitation of this study is the use of data sources collected from available administrative records which may have quality issues and lack of opportunity. Unfortunately, data on HPV test positivity, on treatment to premalignant lesions, and diagnostic evaluation were not available. Additionally, our data does not include information on CC-related hospital admissions, and thus does not cover the most negative outcomes (death or continuous in-patient treatment during the observation period). A specific limitation was the use of single center data for analysis of the impact at the level of tertiary prevention, since INCAN does not necessarily reflect what happens in all specialized hospitals in the health sector. However, given that the regulations of cancer programs are similar for the health sector and considering that it is one of the main resolutive hospitals in the CC and with the largest budget, possibly the impact of the epidemic is greater in the hospitals with less infrastructure.

In conclusion, the COVID-19 pandemic had a direct impact on CC prevention efforts at all levels in Mexico. This study documents the deterioration in the performance of the CC prevention program by demonstrating the adverse impacts of the pandemic on effectiveness and access to health services because of a significant reduction in the number of HPV vaccines applied and the lower number of patients attending first-time and recurrent colposcopy exams. The impact on the program’s efficiency is also evidenced by the increase in the proportion of cases that started treatment more than 8 weeks after diagnosis and the lower proportion of CC cases detected at an early clinical stage.

Therefore, improving the performance of CC prevention program is crucial to reduce delays in vaccination, to achieve long-term reduction in the incidence of HPV infection, guarantee follow-up of positive cases, promote early detection of invasive cancers, timely initiation of treatment, and to promote disease-free survival. Among other measures, home vaginal self-sampling could offer options for CC prevention and treatment to women with restricted access to health services. These strategies should not only aim to fill the unmet needs created by the pandemic, but also eliminate negative and unnecessary aspects of care for this disease.

In this context, we propose some recommendations that include improving the quality of processes at all three levels of care; for example, improving epidemiological surveillance systems by establishing cancer registries; expand the reach of single-dose immunization; self-collection of vaginal samples for timely molecular diagnostics; early detection in older women; and in according to IARC Handbook, switching from conventional cytology to liquid-bases samples to do HPV as primary screening with cytology triage and typing of the same sample, which could have a large impact on efficacy and maybe on compliance to follow up (58).

## Data availability statement

The datasets presented in this study can be found in online repositories. The names of the repository/repositories and accession number(s) can be found in the article/Supplementary Material.

## Author contributions

Conceptualization, KT-P and AC-V. Data curation, LP-M, JH-A and AQ-S. formal analysis, KT-P, LP-M, JH-A and AQ-S. Investigation, KT-P, AC, CA-F, RU, and VM-M. Methodology, KT-P, AC, LP-M, JH-A and AQ-S. Project administration, KT-P. Resources, LP-M, JH-A, AQ-S, TG-C, EA-B, DI-O and LC-P. Software, LP-M, JH-A and AQ-S. Supervision, KT-P. Validation, KT-P and AC. Visualization, LP-M, JH-A and AQ-S. Writing—original draft, KT-P, AC-V, LP-M, JH-A, AQ-S, CA-F, S-RU-S and VM-M. Writing—review and editing, KT-P, AC-V, LP-M, JH-A, AQ-S, S-RU-S, and VM-M. All authors revised the manuscript. All authors contributed to the article and approved the submitted version.
